# Recovery of Li_2_CO_3_ from Spent LiFePO_4_ by Using a Novel Impurity Elimination Process

**DOI:** 10.3390/molecules28093902

**Published:** 2023-05-05

**Authors:** Wen-Lan Chen, Chi Chen, Hao Xiao, Cheng-Wei Chen, Dan Sun

**Affiliations:** 1 CAS Key Laboratory of Design and Assembly of Functional Nanostructures and Fujian Provincial Key Laboratory of Nanomaterials, Fujian Institute of Research on the Structure of Matter, Chinese Academy of Sciences, Fuzhou 350002, Chinaxmchenchi@fjirsm.ac.cn (C.C.);; 2 College of Chemistry and Materials, Fujian Normal University, Fuzhou 350007, China; 3 Xiamen Institute of Rare Earth Materials, Haixi Institutes, Chinese Academy of Sciences, Xiamen 361021, China; 4 Xiamen Key Laboratory of Rare Earth Photoelectric Functional Materials, Xiamen 361021, China; 5 College of Chemistry, Fuzhou University, Fuzhou 350108, China

**Keywords:** battery material recycle, spent LiFePO_4_, Li_2_CO_3_, alkali, impurity elimination

## Abstract

The large-scale implementations of lithium iron phosphate (LFP) batteries for energy storage systems have been gaining attention around the world due to their quality of high technological maturity and flexible configuration. Unfortunately, the exponential production of LFP batteries is accompanied by an annual accumulation of spent batteries and a premature consumption of the lithium resource. Recycling souring critical battery materials such as Li_2_CO_3_ is essential to reduce the supply chain risk and achieve net carbon neutrality goals. During the recovery of Li_2_CO_3_, impurity removal is the most crucial step in the hydrometallurgy process of spent LiFePO_4_, which determines the purity of Li_2_CO_3_. By investigating and comparing the results of impurity elimination from the purified Li^+^-containing liquids with strong and weak alkalis under identical pH conditions, respectively, a strategy based on an alkali mixture has been proposed. The purified Li^+^-containing liquid was, thereafter, concentrated and sodium carbonate was added in order to precipitate Li_2_CO_3_. As a result, a high purity Li_2_CO_3_ (99.51%) of battery grade was obtained. LiFePO_4_ prepared with the recovered Li_2_CO_3_ and FePO_4_ as raw materials also displayed a comparative high capacity and stable cycle performance to the commercial product and further verified the electrochemical activity of the recovered materials.

## 1. Introduction

Lithium-ion batteries (LIBs) have the advantages of a high cycling stability, high specific energy, stable discharge voltage, and small volume [1,2], which make them widely applicable for use in, e.g., electric vehicles (EV), grid energy storage, and 5G-based stations [3,4]. Of the many commercial cathode materials, lithium iron phosphate (LiFePO_4_, abbreviated as LFP) is considered to be a very reliable material for power and energy storage batteries, which is due to its higher lifetime, reduced toxicity, and low cost [5]. According to *The white paper on the development of China’s lithium iron phosphate battery and lithium iron phosphate material industry (2022)* (jointly released by EV Tank, the China YiWei Institute of Economics, and the China Battery Industry Research Institute), the global shipment of LFP batteries reached 172.1 GWh in 2021. EV Tank predicted that the global shipment of LFP batteries will reach 676.7 GWh and 1290.8 GWh by the years 2025 and 2030, respectively. Thus, the extensive use of LFP makes it an urgent issue to develop a reliable recovery solution for spent lithium iron phosphate (SLFP) [6,7]. This is not only advantageous for environmental protection but also alleviates the shortage of lithium resources.

The available recovery technologies for SLFP nowadays mainly include direct regeneration and hydrometallurgy [2,8]. The direct regeneration process is simple, but a low degree of impurities is required for the SLFP to ensure the reusability of the as-synthesized LFP [9,10]. It should be mentioned that SLFP generally contains some common impurity elements such as Na, F, and Al, which come from the other battery components and packaging [11]. Elements like Ni, Ca, Mn, and Mg can also be introduced during the dismantling of the spent battery packs, which further complicates the recycling process. Nowadays, considering economic efficiency, the manual dismantling method is still commonly used in the industrial field, introducing various external impurity elements such as Si, K, etc. Therefore, the industrial recycling of SLFP using the direct regeneration method is difficult to implement. In a sharp contrast, hydrometallurgy has a low energy consumption and bears a high applicability to most commercial SLFP. In the hydrometallurgy process [12,13,14], an acid is usually combined with an oxidation agent to serve as a selective extraction agent, in order to dissolve specific elements into the solution. Previously, Kumar et al. proposed to use an organic acid as a leaching agent to extract lithium from SLFP, and the extraction efficiency could reach as high as 94.83% [15]. Li et al. selectively extracted Li^+^ with a low concentration of an inorganic acid and the extraction efficiency of Li^+^ thereby increased to 96.85% [16]. Furthermore, Peng et al. effectively separated Li^+^ from SLFP by using only an oxidation agent and reached an Li^+^ extraction efficiency of 98% [17]. Jin et al. followed a direct air oxidation-acid extraction method, with a resulting Li^+^ extraction efficiency as high as 99.3% [18].

While continuously improving the Li^+^ extraction efficiency, the complicated impurity system of the raw material still hinders the industrial promotion of recycling SLFP materials [19,20]. These impurities may remain in the final extracted Li^+^-containing liquid and precipitate along with Li^+^, contributing to redundant purification procedures and degrading the quality of the as-obtained Li_2_CO_3_. Hence, it is of great importance to seek a solution to effectively eliminate the impurity elements and produce high-purity lithium products from SLFP. Of many possible approaches to remove the impurities [21,22], the alkali chemical precipitation method has emerged as the most promising route [23,24], which is mainly due to its economic applicability. The alkali chemical precipitation methods usually use sodium hydroxide or ammonia to separate and eliminate the impurities before Li^+^ precipitation [24,25]. Whereas, the elimination efficiencies of different impurities, by adjusting the pH values with different alkalis, have not yet been systematically explored.

In this work, we used a low concentration of H_2_SO_4_ and H_2_O_2_ as a combined leaching agent to selectively extract Li^+^ from SLFP [16]. In addition, NaOH and NH_3_·H_2_O were selected as purifying agents to eliminate impurities from the extracted Li^+^-containing liquid. By studying the elimination efficiencies by using different alkalis, we discovered that using just a single alkali in the purification process was difficult to eliminate all the impurities. Therefore, we have here proposed an impurity removal process that is based on an alkali mixture. A combination of XRD (X-ray diffraction), SEM (scanning electron microscopy), XPS (X-ray photoelectric spectroscopy), and ICP-OES (inductively coupled plasma-optical emission spectrometry) characterization methods was used to analyze the crystalline phase, morphology, valence state, and impurity composition of the recovered Li_2_CO_3_ material. It can be concluded that the recovered Li_2_CO_3_ that has been prepared from a Li^+^-containing liquid, which has been purified using an alkali mixture, possessed much higher purity. The electrochemical activities of the recovered Li_2_CO_3_ and FePO_4_ were also examined by fabricating LFP with commercial precursors, respectively, and the as-obtained LFP delivered comparative capacity and rate capability compared to that prepared from commercial precursors. These results may provide a new direction for industrial recycling of spent lithium iron phosphate in the preparation of lithium carbonate.

## 2. Results and Discussion

**Figure 1:**
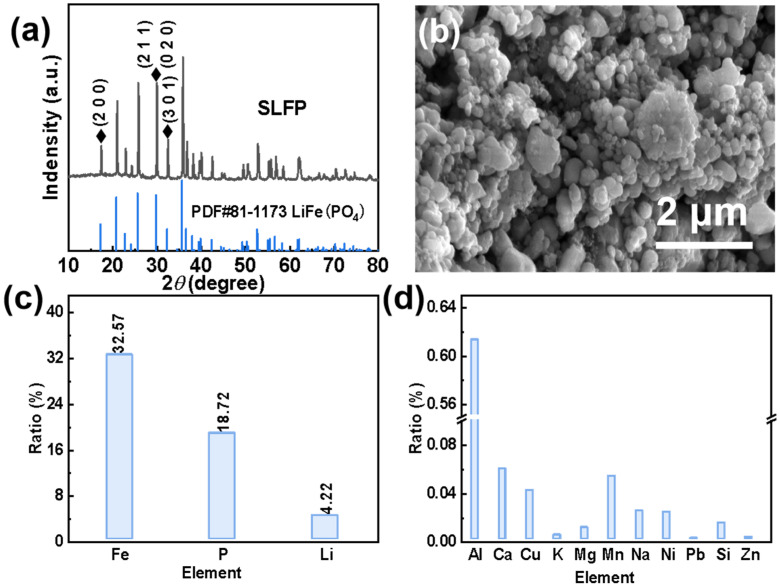
(**a**) XRD pattern, (**b**) SEM image, (**c**) main element contents, and (**d**) impurity contents of the original SLFP material.

In order to effectively extract Li^+^ from SLFP, a comprehensive analysis of SLFP was necessary. Based on the XRD spectrum in Figure 1a, it can be concluded that LiFePO_4_ is the main component of SLFP. As can be seen in the SEM diagram in Figure 1b, SLFP consists of nanometer-sized and coarse particles, and particle surfaces are covered by a layer of conductive carbon “net”. Furthermore, the ratio of the main elements in SLFP is shown in Figure 1c and Table S1. More specifically, the weight ratio of Fe, Li, and P is 32.57%, 4.22%, and 18.72%, respectively. Correspondingly, the molar ratio of Li, Fe, and P is l.04:1:1.04, this slight deviation may be due to the remaining decomposition products of the electrolyte. The proportion of the impurity elements is shown in Figure 1d, and the corresponding date is provided in Table S1.

### 2.1. Selective Extraction from SLFP

The parameters of the extraction process of Li^+^ and from SLFP are critical for accurately dissolving the Li^+^ into the solution, while Fe and P still maintain the solid structure. Three experimental factors are the most decisive for understanding the Li extraction process and need particular investigation. These are the acid concentration (c), the liquid-to-solid (L/S) ratio (i.e., H_2_SO_4_ volume-to-SLFP mass ratio), and the H_2_O_2_/H_2_SO_4_ volume ratio (ψ). In addition, choosing the appropriate temperature (T) will further maximize the selective extraction efficiency of Li^+^, and minimize the collapse of the FePO_4_ framework.

#### 2.1.1. Effect of Acid Concentration

**Figure 2:**
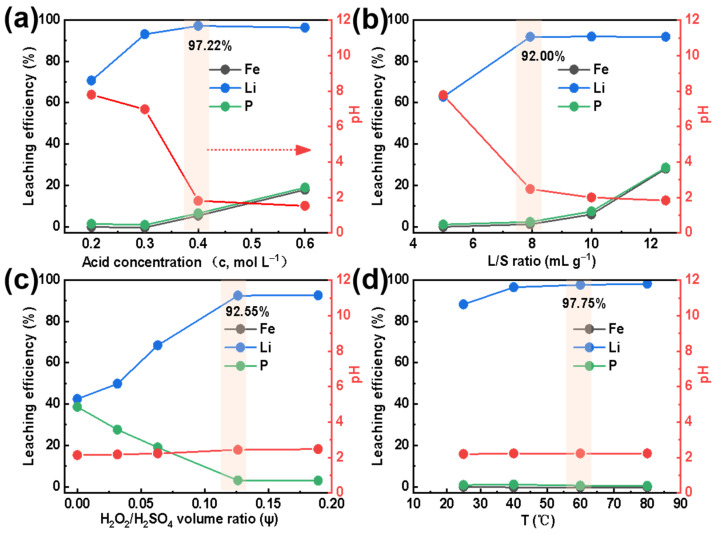
Effect of (**a**) acid concentration (at L/S = 10 mL g^−1^, ψ = 0.10, and T = 60 °C), (**b**) L/S ratio (at c = 0.4 mol L^−1^ and T = 60 °C), (**c**) H_2_O_2_/H_2_SO_4_ volume ratio (at c = 0.4 mol L^−1^, L/S = 7.93 mL g^−1^, and T = 60 °C), and (**d**) temperature (at c = 0.4 mol L^−1^, L/S = 7.93 mL g^−1^, and ψ = 0.13) on the extraction efficiencies (of Li, Fe, and P) and pH of the extracted Li^+^-containing liquid.

Figure 2a shows the extraction efficiency of Li, Fe, and P when using an H_2_SO_4_ concentration ranging from 0.2 mol L^−1^ to 0.6 mol L^−1^. For an H_2_SO_4_ concentration less than 0.3 mol L^−1^, the extraction efficiency of Fe and P is close to 0%. The solubility of all the targeted elements (Li, Fe, P) into the solution grows with increasing H_2_SO_4_ concentration. Peculiarly, when the H_2_SO_4_ concentration equals 0.4 mol L^−1^, the extraction efficiency of Fe and P increases to about 5%, while it reaches the highest value, 97.22% of Li. Due to sufficient H^+^ in the solution and the strong interaction between H and Li, almost all lithium within SLFP has been released from its structural Fe-P-O framework [26]. Since FePO_4_ will be easily dissolved in a strong acidic solution [27,28], to improve the extraction conditions, all experiments have been conducted with a 0.4 mol L^−1^ sulfuric acid.

#### 2.1.2. Effect of Liquid-to-Solid Ratio

Figure 2b exhibits the trend in extraction efficiency for Li, Fe, and P with a L/S value ranging from 5 to 12.5 mL g^−1^. The results indicate that when the liquid-to-solid ratio is less than 7.93 mL g^−1^, the extraction efficiency of Fe and P is close to 0%, and the extraction efficiency of Li is only about 60%. Interestingly, when the L/S ratio is equal to 7.93 mL g^−1^, the extraction efficiency of Fe and P has only increased by about 2%, while it reaches 92.00% for Li. When the L/S ratio is further increased, the extraction efficiency of Fe and P is continuously improved. However, the Li content remains constant. Therefore, a reasonable L/S ratio can ensure the SLFP material is completely dispersed in the extraction agent, which improves the proton transfer speed between the solid and liquid phases and guarantees a high extraction efficiency [29,30]. On the other hand, it will also reduce the processing costs and duration of the concentration process. In order to obtain a higher extraction efficiency and a larger amount of treated SLFP, an L/S value of 7.93 mL g^−1^ was selected for all extraction experiments in this work.

#### 2.1.3. Effect of H_2_O_2_/H_2_SO_4_ Volume Ratio

Figure 2c reveals the effect of the H_2_O_2_/H_2_SO_4_ volume ratio on the Li, Fe, and P extraction. When the volume fraction of H_2_O_2_ increases from 0 to 0.13, the Li extraction efficiency increases from 40% to 92.55%. On the contrary, the extraction efficiency of Fe and P decreases from 40% to 0%. Obviously, it is an undisputed fact that H_2_O_2_ has a major effect on the selective Li extraction. This phenomenon can be explained by the fact that LiFePO_4_ will at first decompose into Li^+^, Fe^2+^, and PO_4_^3−^ in a certain amount of acidic solution. The ferrous ions will, thereafter, gradually oxidize to Fe^3+^ and react with PO_4_^3−^ (to form a FePO_4_ precipitation) with an increasing H_2_O_2_ amount. Thus, this results in a reduction in the P and Fe content in the extracted Li^+^-containing liquid [16]. However, with a further increase in H_2_O_2_ (to ψ = 0.19), the extraction efficiencies remain constant. Furthermore, the pH value of the extracted solution tends to be stable since LiFePO_4_ has been completely dissolved and Fe^2+^ has been oxidized to Fe^3+^. To control the extraction rate of P and Fe, the H_2_O_2_/H_2_SO_4_ volume ratio has been denoted as ψ = 0.13 in the subsequent experiments.

#### 2.1.4. Effect of Temperature

The influence of temperature on the Li, Fe, and P extraction efficiency was also examined. As displayed in Figure 2d, when the temperature increased from 25 °C to 60 °C, the Li extraction efficiency increased from 88.26% to 97.75%. However, the extraction efficiency of Fe and P were both close to 0%. In addition, the pH value in the extracted solution remained constant. Although it is clear that temperature has a substantial effect on the selective extraction of Li, H_2_O_2_ can be easily decomposed at high temperatures, thus, T = 60 °C had been selected here as the optimal temperature condition.

### 2.2. Purification of the Extracted Li^+^-Containing Liquid with Different Alkalis

**Figure 3:**
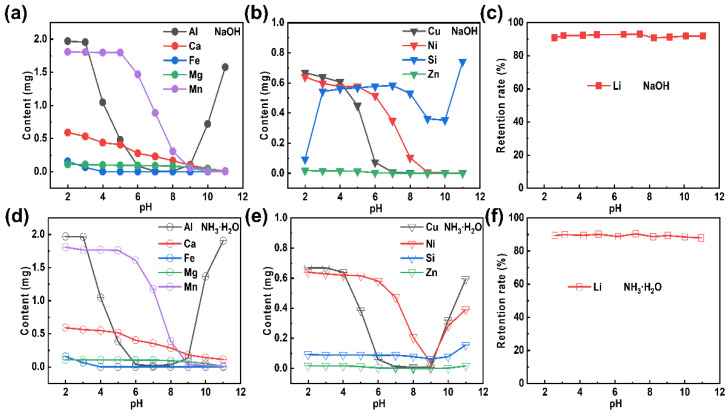
Elemental variation in the purified Li^+^-containing liquid, as obtained by the addition of the purification reagents: (**a**–**c**) NaOH and (**d**–**f**) NH_3_∙H_2_O.

The removal of impurities from the extracted Li^+^-containing liquid is the most critical step to realize in the formation of Li_2_CO_3_ of high purity. Here, an equal volume (50 mL) of extracted Li^+^-containing liquid is used as a mother liquid for pH adjustment to remove impurities. Photos of the purified Li^+^-containing liquid, which has been treated with alkalis (sodium hydroxide and ammonia) at different pH values, are shown in Figure S1 in the Supplementary Information. Almost no precipitation can be observed at a pH value less than 5, while most of the metal ions had precipitated when the pH value continuously increased [31,32]. It can also be seen that the colors of the precipitates became deepened as the pH value increased, which indicated that the impurity ions had been gradually separated out. The extracted Li^+^-containing liquid had thereby become purified. Moreover, the purified Li^+^-containing liquid was characterized by ICP, at a specific pH value, to further explore the condition of the removed impurities. The impurity residues in the purified Li^+^-containing liquid are presented in Table S2 (NaOH) and Table S3 (NH_3_·H_2_O) in the Supplementary Information. The content of Pb is extremely low that it can be ignored. Noteworthy, Na and K are alkali metal elements of high solubility [33,34], and have little effect on the subsequent lithium precipitation to some extent. The results of the LLP_Na_ and LLP_NH_3__ treatments at various pH values (as shown in Figure 3a,d, respectively) can be summarized as following: (Ⅰ) The optimal pH value for the Al removal is 7; (Ⅱ) Fe can be completely removed at a pH value of 4; (Ⅲ) The amount of Ca, Mg, and Mn decreases with the addition of an alkali. However, there are some distinct differences in results for the LLP_Na_ and LLP_NH_3__ treatments (as shown in Figure 3b,e, respectively): (Ⅰ) The Si impurity removal efficiency of LLP_NH_3__ is higher than that of LLP_Na_; (Ⅱ) The contents of Cu, Ni, and Zn gradually decrease during the LLP_Na_ treatment, while they reach a minimum at pH = 9 during the LLP_NH_3__ treatment (followed by a content increase with a continuous increase in the pH value). This can be explained by the complexation reactions that take place at higher concentrations of NH_3_·H_2_O [35,36,37]. Importantly, whether NaOH (Figure 3c) or NH_3_·H_2_O (Figure 3f) is used as a purification reagent, the Li content hardly changes as the pH value varies.

In order to improve the efficiency of the purification process, we proposed and used a combination of two different alkalis for adjusting the pH value of the extracted Li^+^-containing liquid. The contents of the elements in the extracted Li^+^-containing liquid that were purified using the LLP_NaNH_3__ strategy are shown in Table 1. As concluded, most impurities can be removed when using a mixture of alkalis.

### 2.3. Analysis of Recovered Li and Elemental Impurities

**Figure 4:**
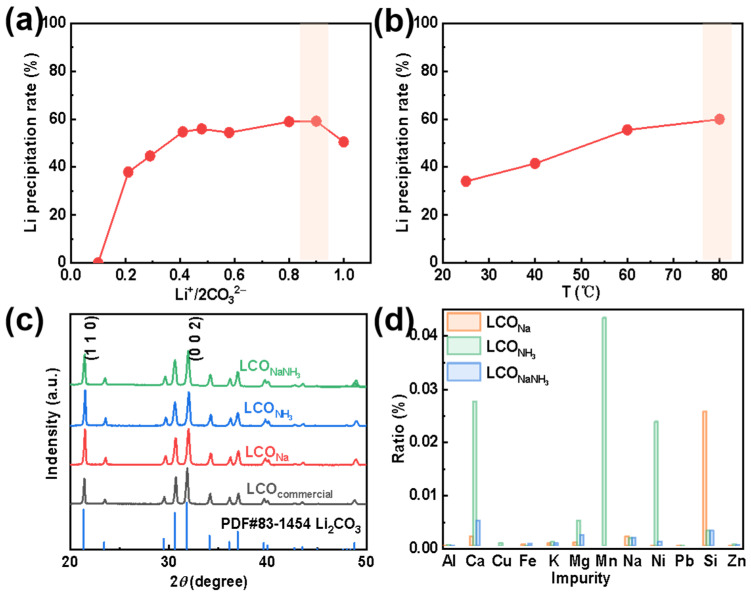
Effects of (**a**) Li^+^/2CO_3_^2−^ and (**b**) temperature on the Li^+^ precipitation rate. (**c**) XRD patterns and (**d**) elemental contents of Li_2_CO_3_ with different purification agents.

After the removal of Fe and other elemental impurities from the extracted Li^+^-containing liquid, using either a strong alkali (NaOH), a weak alkali (NH_3_·H_2_O), or a mixture of alkalis (NaOH&NH_3_·H_2_O), a series of purified Li^+^-containing liquids were obtained. The purified Li^+^-containing liquid was, thereafter, condensed at a temperature below 100 °C to achieve a higher Li^+^-concentration of about 30 g L^−1^. The final step was the addition of Na_2_CO_3_ with a specific concentration to form Li_2_CO_3_ (from here on denoted as LCO). Depending on different types of purification agents, LCO_Na_, LCO_NH_3__, and LCO_NaNH_3__ have been formed. The effects of the molar ratio of Li^+^/2CO_3_^2−^ and reaction temperature on the precipitation rate of LCO are shown in Figure 4a,b, respectively. (In the form of Na_2_CO_3_ and Li_2_CO_3_, two Li^+^ ions are couples with one CO_3_^2−^). Here, the total amount of Li^+^ in the concentrated solution is settled. As can be seen in Figure 4a, the Li^+^ precipitation rate increases initially to a maximum and then decreases with an increase in Li^+^/2CO_3_^2−^. It is worth mentioning here, when Li^+^/2CO_3_^2−^ is very large, which indicates a small amount of Na_2_CO_3_ has been added, insufficient CO_3_^2−^ results in a relatively low Li^+^ precipitation rate. Instead, when Li^+^/2CO_3_^2−^ is very low, the excessive amount of Na_2_CO_3_ may increase the total volume of the solution, leading to a decrease in the Li^+^ concentration, thus declines the Li^+^ precipitation rate.

The solubility of LCO decreases (i.e., the precipitation rate of LCO increases) with an increase in temperature. To obtain a relatively high primary Li recovery rate, the conditions highlighted by an orange-filled rectangle in Figure 4a,b have been used in the forthcoming experiments. Figure 4c displays the XRD patterns of commercial LCO and of the three types of recycled LCO (i.e., LCO_Na_, LCO_NH_3__, and LCO_NaNH_3__). All these four diffraction patterns are consistent with the standard PDF card for crystalline lithium carbonate. SEM analysis of LCO_commercial_ has revealed a morphology content of irregular blocks (Figure S2a in the Supplementary Information); while LCO_Na_ and LCO_NH_3__ possess a flower-shaped morphology formed by stackings of smooth flakes (Figure S2b,c) [26]. Some small particles can also be observed on LCO_NH_3__, which can be explained by the poor impurity removal efficiency by NH_3_ on Mn, Ca, Mg, and other elements. Moreover, the morphology of LCO_NaNH_3__ turns out be random agglomerations of LCO flakes (Figure S2d).

To further analyze the impurities contained in LCO, the mass fractions of these metals have been characterized using ICP-OES. As shown in Figure 4d, it is obvious that LCO recovered from LLP_Na_ and LLP_NH_3__ both have a high content of different impurity elements. Particularly, Si cannot be effectively eliminated by LLP_Na_ and the remaining Mn, Ni, and Ca amounts far exceed the other elimination processes by LLP_NH_3__. However, the purity of LCO_NaNH_3__ has reached 99.51%, which meets the requirements of battery grade lithium carbonate (Table S4 in the Supplementary Information). We have, in addition, utilized the XPS characterized method for further analysis of different LCO surfaces (Figure S3). The C 1s spectrum is divided into four peaks (Figure S3a), which is corresponding to C=O and O=C–O from lithium carbonate (289.8 eV) [38], C–O (288.1 eV), C–C (285.9 eV), and C=C (284.8 eV) bonds (with surface binding C atoms) [39], respectively. In the O 1s spectrum, the pink-filled peak represents the O 1s peak of lithium carbonate (at about 531.6 eV), and the broad peak at 532.3 eV corresponds to oxygen that may have been chemically adsorbed to the surface (such as O–H or O_2_^−^) [40]. Finally, the Li 1s spectrum has been fitted as a single peak with a maximum at about 55.1 eV [41], which is close to previously reported values for lithium carbonate.

### 2.4. Recovery and Analysis of the Extraction Residue

We have also calcined (i.e., sintered) the residues from the selective extraction process at a temperature of 700 °C for 3 h, in order to analyze the chemical status of iron ions. The crystalline structure of this product, before and after sintering, was characterized using XRD (Figure S4a) and the morphology was analyzed by SEM (Figure S4b,c). The XRD spectrum that is presented in Figure S4a shows that the obtained iron products are almost identical with the PDF card of FePO_4_ (PDF#84-0876). Furthermore, Figure S4b,c in the Supplementary Information show FePO_4_ particles with a relatively uniform distribution, both before and after sintering. This result is consistent with the results of previous research, which further verify the extraction process described above [42]. In addition, the composition analysis of recovery FePO_4_ is shown in Table S5.

### 2.5. LFP Material Regeneration and Tests of Its Performance

**Figure 5:**
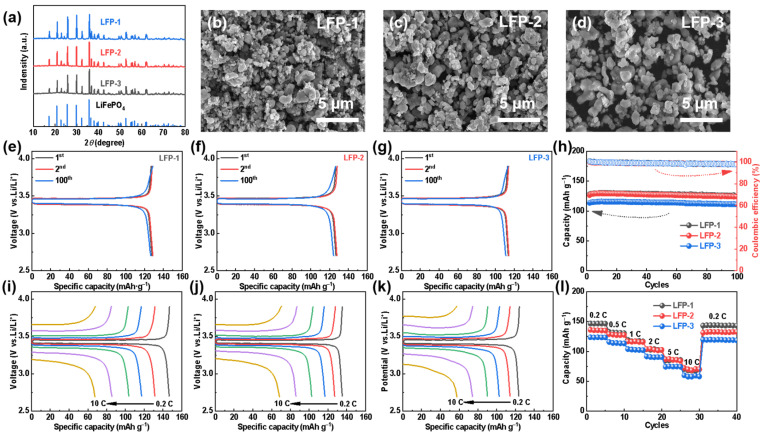
For different LiFePO_4_ materials: (**a**) XRD spectra, (**b**–**d**) SEM diagrams, (**e**–**g**) typical charge—discharge curves, (**h**) cycle performances at 0.5 C, (**i**–**k**) rate capabilities, and (**l**) rate performances. (**b**,**e**,**i**) LFP-1, (**c**,**f**,**j**) LFP-2, and (**d**,**g**,**k**) LFP-3.

LiFePO_4_ has been prepared by using carbothermal reduction with LCO as a lithium source and FP as a phosphorus and iron source, in order to discuss the electrochemical activities of the recovered materials. Specifically, LFP-1, LFP-2, and LFP-3 refers to synthesized LFP using both commercial FePO_4_ and Li_2_CO_3_, commercial FePO_4_ and recovered Li_2_CO_3_ (LCO_NaNH_3__), and recovered FePO_4_ and commercial Li_2_CO_3_ as raw materials, respectively. The XRD of all the regenerated LFP samples match well with the PDF card of LiFePO_4_ (Figure 5a), which strongly indicates the formation of pure-phased LFP. The morphologies of LFP-1, LFP-2, and LFP-3 were also investigated by SEM. Figure 5b–d show a relatively characteristic homogeneous structure for all the samples. The size of the primary particles of all the LFP obtained is micro-or nano-sized, although the aggregation of the primary particles is partially observed. Among them, LFP-1 displays the smallest size of the primary particles, which is due to the uniform and smaller particle size of the commercial precursors.

The electrochemical properties of the LFP samples were characterized by coin cells employing a lithium plate as a counter electrode. As can be seen in Figure 5e–h, all the LFPs undergo a stable cycling over 100 cycles while maintaining high coulombic efficiency at 0.5 C. The initial discharge capacities of the three synthetic LFPs at were 127.8 (LFP-1), 126.7 (LFP-2), and 113.6 (LFP-3) mAh g^−1^, respectively. Since the recycled FP has not been further purified and milled to optimize the particle size, LFP-3 exhibits much lower cell capacities. In addition, the regenerated LFPs also deliver excellent rate capabilities, as shown in Figure 5i–l. As the current rates from 0.2 C to 10 C, the capacities of LFP-2 are 135.2, 127.2, 116.7, 102.7, 85.8, and 68.4 mAh g^−1^, respectively, comparable to those of LFP-1. However, when the current density returns to 0.2 C, the difference in the discharge capacity indicates the less structural stability of LFP-2 than LFP-1, which can be attributed to the lack of regranulation of the recycled LCO particles.

## 3. Experimental Section

### 3.1. Materials and Characterization

The SLFP materials were supplied by Xiamen Hithium Energy Storage Technology Co., Ltd., Xiamen, China. Furthermore, H_2_SO_4_, H_2_O_2_ (30%), Na_2_CO_3_, NH_3_·H_2_O, NaOH, FePO_4_, and Li_2_CO_3_ were purchased from Xiamen Chenhong Environmental Protection Technology Co., Ltd., Xiamen, China. All solutions were prepared using ultrapure water (resistivity = 18.25 MΩ.cm, UPT-ΙΙ-20T; Sichuan—ULUPURE Ultrapure Technology Co., Ltd., Chengdu, China).

X-ray diffraction spectroscopy (XRD; Miniflex 600) was used to characterize the crystalline phases of the solid samples. Furthermore, scanning electron microscopy (SEM; Apreo S LoVac) was used to analyze the morphology of the solid samples and X-ray photoelectron spectroscopy (XPS; Axis Supra) was used to analysis the valence states of recovered Li_2_CO_3_. Inductively coupled plasma-optical emission spectrometry (ICP-OES; ULTIMA 2) was used to measure the elemental contents in SLFP and Li_2_CO_3._ In addition, the electrochemical properties of the prepared lithium iron phosphate (LiFePO_4_) were tested using a LAND battery test workstation.

### 3.2. Experimental Procedure

#### 3.2.1. Analysis of the Components in SLFP

**Figure 6:**
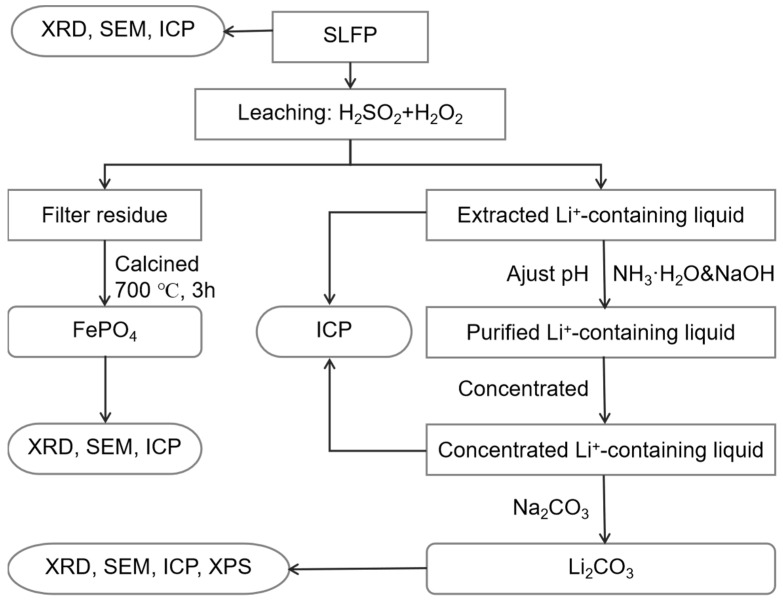
The schematic configuration of SLFP recycling process.

A flowchart of the integrated process of the Li_2_CO_3_ and FePO_4_ recovery from SLFP is shown in Figure 6. Roughly, the SLFP material was initially analyzed using XRD and ICP-OES to get a comprehensive understanding of the composition and elemental contents of the raw material. Incidentally, the possibility of SLFP being in the discharge state has also been investigated using XRD [43]. Based upon these parameters, the SLFP material was thereafter completely leached out using a mixture of H_2_SO_4_ and H_2_O_2_ of various concentrations. The leached solution was analyzed using ICP-OES in the measurement of the elemental content.

#### 3.2.2. Selective Extraction from SLFP

The process of Li^+^ extraction from SLFP is theoretically presented in Equation (1).

(1) $$2{\mathrm{LiFePO}}_{4}+2H^{+}+{\;H}_{2}O_{2}\;\to \;2{\mathrm{Li}}^{+}+2{\mathrm{FePO}}_{4}+2H_{2}O\;$$

Experimentally, SLFP has been mixed with different concentrations of H_2_SO_4_ (0.2 mol L^−1^ to 0.6 mol L^−1^) and contents of H_2_O_2_ (0 to 0.19). In addition, there was a variation in the liquid-to-solid ratio (L/S) (5 mL g^−1^ to 12.5 mL g^−1^). The temperature was, subsequently, adjusted to optimize the Li^+^ extraction process. This process is a reduction-oxidation reaction that has been widely used to selectively extract Li^+^ from SLFP [8,16].

#### 3.2.3. Purification of the Extracted Li^+^-Containing Liquid

In order to prepare Li_2_CO_3_ of a higher purity, it is necessary to remove the impurities from the extracted Li^+^-containing liquid. We have used different types of alkali solutions (NaOH, NH_3_·H_2_O, and NaOH&NH_3_·H_2_O) for this purpose. These purification processes are from here on defined as LLP_Na_ (Li-containing Liquid Purification), LLP_NH_3__, and LLP_NaNH_3__, respectively. The impurity contents of the different purified Li^+^-containing liquids were analyzed using ICP-OES.

#### 3.2.4. Precipitation of Li_2_CO_3_ and Analysis of Its Impurities

In this step, the purified Li^+^-containing liquid was first concentrated up to 30 g L^−1^ and followed by the introduction of Na_2_CO_3_ so as to precipitate Li^+^ in the form of Li_2_CO_3_. This precipitation process can be expressed as:

(2) $$2{\mathrm{Li}}^{+}+{\;\mathrm{CO}}_{3}{}^{2-}\;\to {\mathrm{Li}}_{2}{\mathrm{CO}}_{3}\left(s\right)$$

The crystalline phase and morphology of Li_2_CO_3_ was, thereafter, characterized using XRD and SEM. Furthermore, ICP-OES and XPS were used to measure the contents of impurity elements and the valence states of Li_2_CO_3_.

#### 3.2.5. Recycling and Characterization of FePO_4_

After the extraction and filtering of extracted Li^+^-containing liquid, the filtered residue was dried at 80 °C overnight to remove the liquid of filtered residue and then annealed (i.e., calcined) at 700 °C for 3 h to obtain FePO_4_ (defined as FP). In addition, the filtered residue, before and after annealing, was characterized by using XRD and SEM.

#### 3.2.6. Preparation of Lithium Iron Phosphate and Its Properties

Lithium iron phosphate (LiFePO_4_, defined as LFP) was prepared using a facile carbothermal reduction method, with the reconstructed Li_2_CO_3_ as the lithium source and the reconstructed FePO_4_ as the phosphorus and iron source. From here on, LFP-1 refers to commercial FePO_4_ and commercial Li_2_CO_3_, LFP-2 refers to commercial FePO_4_ and recovered Li_2_CO_3_, and LFP-3 refers to recovered FePO_4_ and commercial Li_2_CO_3_. Furthermore, electrochemical performance of different LFP materials were measured and discussed.

## 4. Conclusions

A simple and effective impurity removal strategy has here been proposed for the preparation of Li_2_CO_3_ from spent LiFePO_4_ using a hydrometallurgical process. Under optimized extraction conditions (c = 0.4 mol L^−1^, L/S = 7.93 mL g^−1^, ψ = 0.13, and T = 60 °C), a high lithium extraction efficiency with low impurities in the extracted solution could be achieved. The pH value for an optimal impurity removal was determined, and the influence of purification steps on the retention of lithium was further analyzed. Moreover, by comparing the qualities of different purifying agents for Li^+^-containing liquids, it was demonstrated that LCO recovered by using an alkali mixture meets the battery grade. Regenerated LiFePO_4_ prepared using recycled LCO_NaNH3_ displayed excellent rate capability, an initial discharge capacity of 126.7 mAh g^−1^, and a capacity retention of 98.02% over 100 cycles at 0.5 C, verifying the electrochemical activity and high purity of recycled LCO_NaNH3_. These results may provide guidance for future recycling of waste battery materials in the industry.

## Figures and Tables

**Table 1:** Impurity content of a Li^+^-containing liquid, before and after the addition of a purification agent (i.e., NaOH&NH_3_·H_2_O).

Liquid	Mass of Li and Elemental Impurities (mg)												
	Al	Ca	Cu	Fe	K	Mg	Mn	Na	Ni	Pb	Si	Zn	Li
Extracted Li^+^-containing liquid	1.97	0.59	0.67	0.16	0.10	0.11	1.81	0.42	0.64	0.00	0.09	0.02	103.27
Purified Li^+^-containing liquid	0.05	0.10	0.00	0.00	0.08	0.00	0.00	11.30	0.00	0.00	0.18	0.00	102.10

## Data Availability

Not applicable.

## Supplementary Materials

The following supporting information can be downloaded at: https://www.mdpi.com/article/10.3390/molecules28093902/s1. Figure S1: The photo of purified Li^+^-containing liquid obtained by adding purification reagent (NaOH and NH_3_·H_2_O). Figure S2: SEM of different lithium carbonate: (a) LCOcommercial, (b) LCO_Na_, (c) LCO_NH3_, and (d) LCO_NaNH3_. Figure S3: XPS spectrum of (a) C 1 s, (b) O 1 s, (c) Li 1 s in LCO_Na_, LCO_NH3_, and LCO_NaNH3_. Figure S4: (a) XRD and SEM (b) before sintering and (c) after sintering of FePO_4_. Table S1: ICP-OES analysis of the SLFP. Table S2: The result data of purified Li^+^-containing liquid by adding purification reagent (NaOH). Table S3: The result data of purified Li^+^-containing liquid by adding purification reagent (NH_3_·H_2_O). Table S4: Composition analysis of different Li_2_CO_3_. Table S5: Composition analysis of the FePO_4_.

## Sample Availability

Samples of the compounds FePO_4_ and Li_2_CO_3_ are available from the authors.
