# Supplementary material for: Recovery of Li2CO3 from Spent LiFePO4 by Using a Novel Impurity Elimination Process
Source: Molecules. 2023 May 5;28(9):3902. doi: 10.3390/molecules28093902 (PMC10180280; doi:10.3390/molecules28093902)
Supplement: Supplementary file 1 [file molecules-28-03902-s001.zip › molecules-2361561-supplementary.pdf]

Recovery of Li_2CO_3 from spent $LiFePO_4$ by using a novel impurity elimination process

Wen-Lan Chen,[1,2,3,4] Chi Chen,[1,3,4] Hao Xiao,[1,3,4,5] Cheng-Wei Chen,[1,3,4,*] Dan Sun[1,3,4,*]

[1] CAS Key Laboratory of Design and Assembly of Functional Nanostructures, and Fujian Provincial Key Laboratory of Nanomaterials, Fujian Institute of Research on the Structure of Matter, Chinese Academy of Sciences, Fuzhou 350002, China

[2] College of Chemistry and Materials, Fujian Normal University, Fuzhou 350007, China

[3] Xiamen Institute of Rare Earth Materials, Haixi Institutes, Chinese Academy of Sciences, Xiamen 361021, China

[4] Xiamen Key Laboratory of Rare Earth Photoelectric Functional Materials, Xiamen 361021, China

[5] College of Chemistry, Fuzhou University, Fuzhou 350108, China

E-mail address: xmsundan@fjirsm.ac.cn

*Corresponding author

E-mail address: chenchengwei@fjirsm.ac.cn (C. Chen), xmsundan@fjirsm.ac.cn(D. Sun)

Supporting information

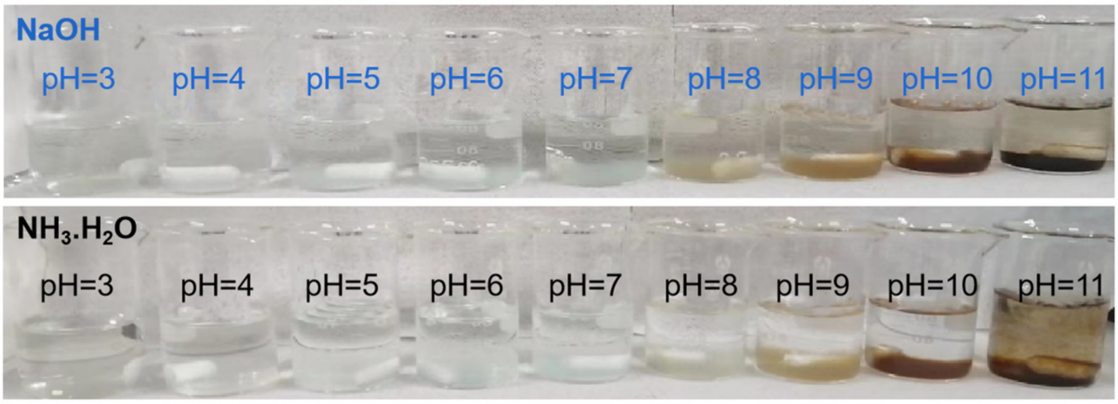

Figure S1. The photo of purified Li^+-containing liquid obtained by adding purification reagent (NaOH and $NH_3 \cdot H_2O$).

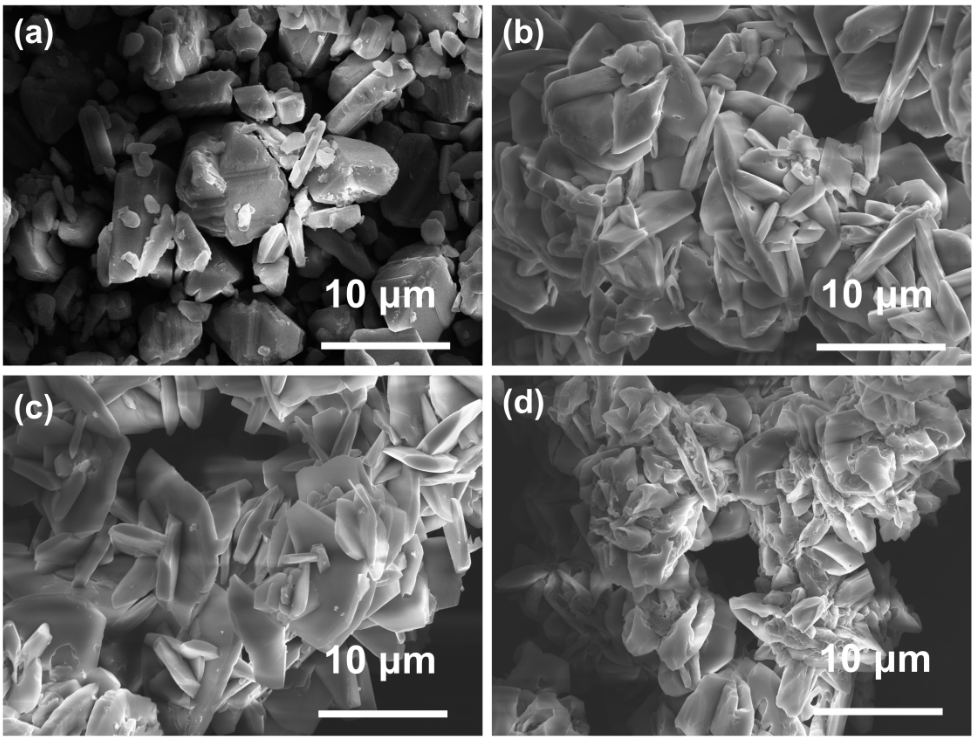

Figure S2. SEM of different lithium carbonate: (a) $LCO_{commercial}$, (b) LCO_{Na}, (c) LCO_{NH_3} and (d) LCO_{NaNH_3}.

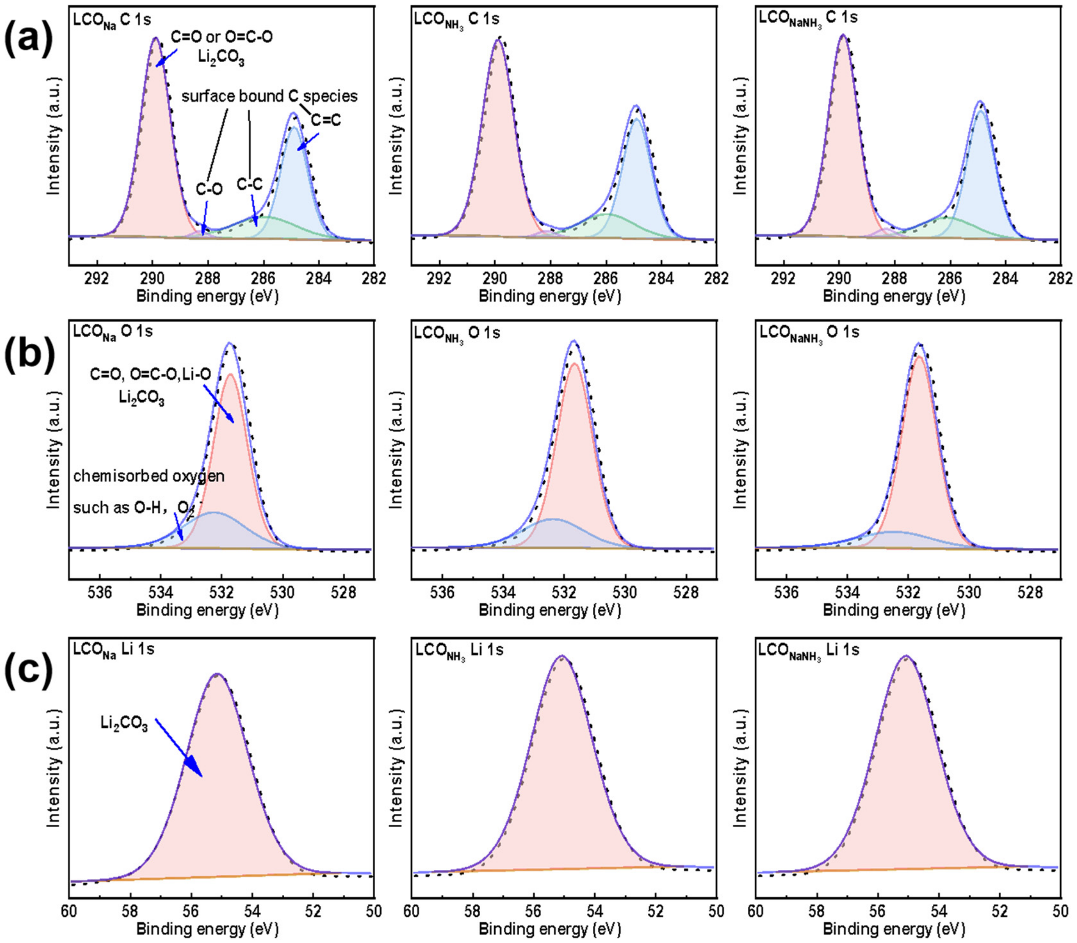

Figure S3. XPS spectrum of (a) C 1s, (b) O 1s, (c) Li 1s in LCO_{Na}, LCO_{NH_3} and LCO_{NaNH_3}.

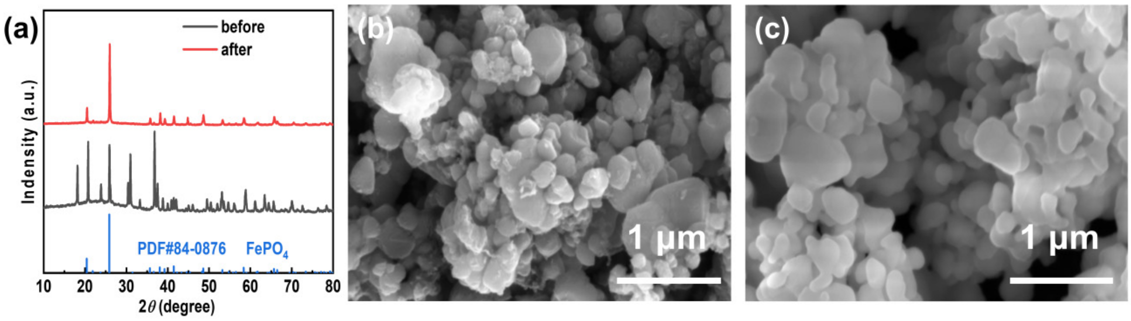

Figure S4. (a) XRD and SEM (b) before sintering and (c) after sintering of $FePO_4$.

Table S1 ICP-OES analysis of the SLFP.

Elements	Fe	P	Li	Al	Ca	Cu	K	Mg	Mn	Na	Ni	Pb	Si	Zn
Mass ration (%)	32.57	18.72	4.22	0.613	0.059	0.041	0.004	0.010	0.053	0.024	0.023	0.001	0.014	0.002

Table S2. The result data of purified Li^{+}-containing liquid by adding purification reagent (NaOH).

pH	Mass of elemental impurities (mg)											
	Al	Ca	Cu	Fe	K	Mg	Mn	Na	Ni	Pb	Si	Zn
2	1.97	0.59	0.67	0.16	0.10	0.11	1.81	0.42	0.64	0.00	0.09	0.02
3	1.96	0.58	0.64	0.03	0.08	0.11	1.80	5.34	0.60	0.00	0.54	0.02
4	1.05	0.49	0.61	0.00	0.08	0.10	1.80	8.10	0.58	0.00	0.56	0.02
5	0.48	0.46	0.45	0.00	0.10	0.10	1.80	9.01	0.58	0.00	0.57	0.01
6	0.08	0.33	0.07	0.00	0.10	0.09	1.47	10.34	0.51	0.00	0.58	0.00
7	0.01	0.28	0.01	0.00	0.11	0.09	0.89	11.56	0.35	0.00	0.58	0.00
8	0.01	0.22	0.00	0.00	0.11	0.08	0.31	12.37	0.10	0.00	0.53	0.00
9	0.10	0.15	0.00	0.00	0.12	0.07	0.01	12.65	0.01	0.00	0.36	0.00
10	0.72	0.09	0.00	0.00	0.12	0.04	0.01	12.99	0.00	0.00	0.35	0.00
11	1.58	0.06	0.00	0.00	0.13	0.00	0.00	14.35	0.00	0.00	0.74	0.00

Table S3. The result data of purified Li^+-containing liquid by adding purification reagent ($NH_3 \cdot H_2O$).

pH	Mass of elemental impurities (mg)											
	Al	Ca	Cu	Fe	K	Mg	Mn	Na	Ni	Pb	Si	Zn
2	1.97	0.59	0.67	0.16	0.10	0.11	1.81	0.42	0.64	0.00	0.09	0.02
3	1.97	0.56	0.67	0.06	0.05	0.11	1.77	0.37	0.63	0.00	0.09	0.02
4	1.05	0.55	0.64	0.00	0.10	0.11	1.77	0.40	0.62	0.00	0.09	0.02
5	0.38	0.52	0.39	0.00	0.08	0.11	1.76	0.40	0.62	0.00	0.09	0.02
6	0.03	0.40	0.06	0.00	0.08	0.10	1.61	0.40	0.58	0.00	0.09	0.00
7	0.02	0.36	0.01	0.00	0.08	0.10	1.18	0.40	0.47	0.00	0.09	0.00
8	0.03	0.29	0.01	0.00	0.07	0.10	0.39	0.39	0.21	0.00	0.08	0.00
9	0.13	0.18	0.01	0.00	0.07	0.08	0.04	0.39	0.04	0.00	0.06	0.00
10	1.37	0.14	0.32	0.00	0.09	0.05	0.02	0.35	0.28	0.00	0.08	0.00
11	1.91	0.11	0.59	0.00	0.10	0.01	0.01	0.37	0.39	0.00	0.16	0.02

Table S4. Composition analysis of different Li_2CO_3.

Impurity	Ratio of Li_2CO_3 (%)		
	LCO_{Na}	LCO_{NH_3}	LCO_{NaNH_3}
Al	0.0001	0.0002	0.0001
Ca	0.0018	0.0274	0.0048
Cu	0.0000	0.0006	0.0000
Fe	0.0003	0.0001	0.0004
K	0.0005	0.0008	0.0005
Mg	0.0007	0.0048	0.0021
Mn	0.0000	0.0433	0.0000
Na	0.0018	0.0015	0.0016
Ni	0.0001	0.0236	0.0008
Pb	0.0001	0.0001	0.0000
Si	0.0255	0.0029	0.0029
Zn	0.0001	0.0003	0.0002
Li_2CO_3	99.35	97.18	99.51

Table S5 Composition analysis of the $FePO_4$.

Test item	Fe	P	Fe:P	Al	Ca	Cu	K	Mg	Mn	Na	Ni	Pb	Si	Zn
Mass ratio (%)	36.42	20.70	0.98	0.0718	0.0137	0.0346	0.0061	0.0079	0.0163	0.0429	0.0141	0.0034	0.0307	0.0057
